# Birth ball for pregnant women in labour research protocol: a multi-centre randomised controlled trial

**DOI:** 10.1186/s12884-019-2305-8

**Published:** 2019-05-06

**Authors:** May Pui Shan Yeung, Katrina Wai Kay Tsang, Benjamin Hon Kei Yip, Wing Hung Tam, Wan Yim Ip, Florence Wai Lei Hau, Margaret Kit Wah Wong, Judy Wai Ying Ng, Sau Ha Liu, Sophia Shu Wing Chan, Chi Kin Law, Samuel Yeung Shan Wong

**Affiliations:** 1 Jockey Club School of Public Health and Primary Care (JCSPHPC), The Chinese University of Hong Kong (CUHK), Prince of Wales Hospital, Sha Tin, Hong Kong China; 2 Department of Obstetrics & Gynaecology, The Chinese University of Hong Kong, Prince of Wales Hospital, Hong Kong, China; 3 New Asia College, The Chinese University of Hong Kong, Hong Kong, China; 4 School of Midwifery, Prince of Wales Hospital, Hong Kong, China; 5 Department of Obstetrics & Gynaecology, Prince of Wales Hospital, Hong Kong, China; 6 Department of Obstetrics & Gynaecology, Princess Margaret Hospital, Hong Kong, China; 7 Department of Obstetrics & Gynaecology, Pamela Youde Nethersole Eastern Hospital, Hong Kong, China; 8 Centre for Applied Health Economics (CAHE), School of Medicine, University of Griffith, Brisbane, Australia

**Keywords:** Birth ball, Non-pharmacological, Pain relief, Pregnant women, RCT

## Abstract

**Background:**

Birth ball is one of the non-pharmacologic pain relief methods to help mothers cope with the labouring process. A randomised controlled trial (RCT) is conducted to evaluate the effectiveness, safety and harm of birth ball use by pregnant women in labour compared to treatment as usual group.

**Methods:**

A prospective multi-centre randomised controlled trial (RCT) will be conducted in Obstetrics and Gynaecological units of five public hospitals in Hong Kong, China. Data will be collected from March 2016 onward for 2 years. The target population is Chinese women with an uncomplicated singleton pregnancy at gestational age of 37 to 42 weeks. Participants are randomised based on parity (nulliparous and multiparous) and type of labour onset (spontaneous and induced). Women in the intervention group are actively offered and taught how to use a birth ball; those in the control group receive the usual midwifery care. The target sample size is 512. The primary outcome measures are maternal pain intensity, satisfaction with pain relief, sense of control in labour, assisted delivery and satisfaction with childbirth experience. Labour pain relief is measured by visual analogue scale (VAS). Other outcomes will be measured through four different validated questionnaires. To control for potential cluster effects, a linear mixed model will be used. An intention-to-treat analysis is adopted and performed by researchers unknown to subjects’ group allocation.

**Discussion:**

Results will provide rigorous scientific evidence for policy development and practice. We are using stratified randomisation according to potential confounders of parity and type of labour onset to give four possible combinations. If the results are favourable, it will facilitate systematic implementation to promote birth ball use for women in labour.

**Trial registration:**

Chinese Clinical Trial Register (ChiCTR), Registration number: ChiCTR-IIC-16008275, Date of registration 12 April 2016 (retrospectively registered), Date of enrolment of the first participant to the trial 1 March 2016.

## Background

Pain relief in labour was identified by an overwhelming majority (78%) of consumer groups and researchers to be one of the most important topics related to pregnancy and childbirth [1]. Given the frequent adverse effects of pharmacological analgesia and the healthcare cost associated with their complications, there is an incentive to use non-pharmacologic pain relief methods for pregnant women [2, 3]. Non-pharmacological methods can ease sensations of pain in a number of ways, by promoting women’s wellbeing, comfort, and sense of control in labour [4]; and the birth ball is one of the methods to help mothers cope with the labouring process and childbirth.

The birth ball, also called fitball or swiss ball, is a large ball commonly with a diameter of 55 cm or 65 cm. It provides a soft surface for women to sit on or lean against while carrying out simple exercises. Birth ball exercises can directly relieve women’s physical pain by improving pelvic dimensions, mobility and foetal positioning [5–7]. Concurrently, women’s psychosocial wellbeing can be enhanced when they take an active role in their own care, thereby promoting a sense of control over their care and body postures, balance and coordination.

Three local observational studies [8–10] reported strong acceptability and high satisfaction (over 90%) among Chinese women in Hong Kong public hospitals, who used birth balls during labour. Evidence for the use of a birth ball by pregnant mothers and related childbirth outcomes is insufficient. Thus far, there has been one systematic review with meta-analysis and four randomised controlled trials (RCTs) conducted in four countries: Spain, Taiwan, Iran, and Brazil [11–15]. These RCTs have been small, totalling 220 subjects, and thus the true effect size of a birth ball on pain control would be difficult to calculate due to chance and bias.

According to the result of the systematic review, more rigorous RCTs are necessary to evaluate the effect of birth balls on pain relief [11]. Searches in the World Health Organisation International Clinical Trials Registry Platform using keyword ‘birth ball’, returned six RCTs with subjects ranges from 32 to 128 [16]. Large scale clinical trials with adequate statistical power are needed to examine the effects of birth ball use by women in labour on various maternal, neonatal and infant outcomes, and system outcomes such as healthcare cost.

## Methods

A randomised controlled trial (RCT) is designed and the objective is to evaluate the effectiveness, safety and harm of birth ball use by pregnant women in labour compared to treatment as usual group. The research hypothesis is that participants randomised to the birth ball intervention group have better pain relief, higher satisfaction with pain control, higher sense of control in labour and higher satisfaction with childbirth experience, when compared to subjects in control group. This study protocol follows the SPIRIT checklist.

### Study setting

**Fig. 1:**
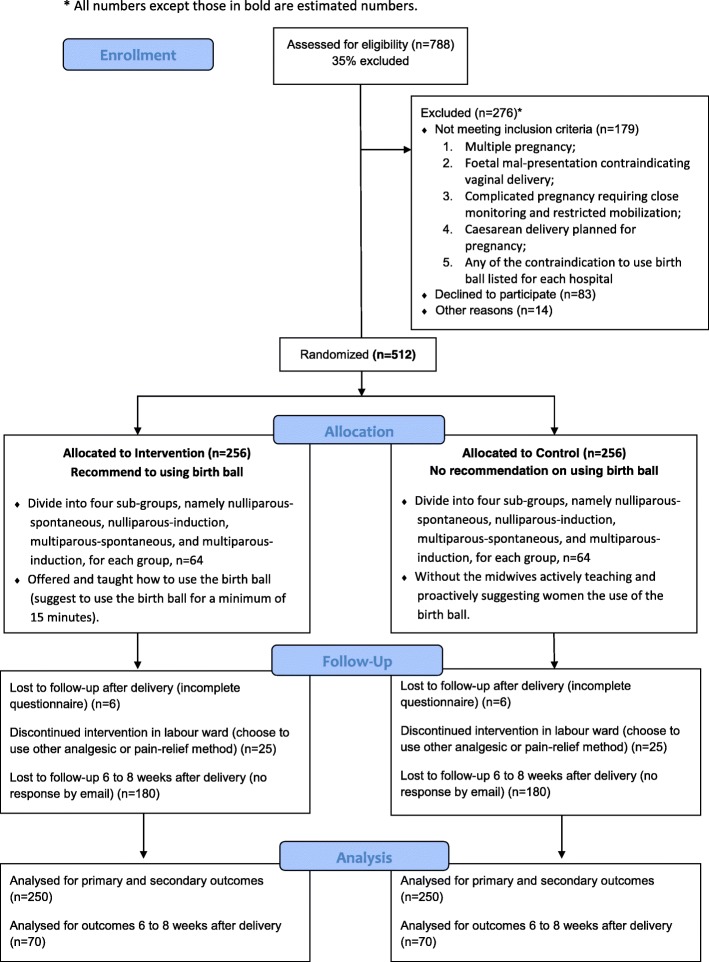
Consort flow diagram of the trial

Recruitment for the study is conducted in all three regions of Hong Kong to enhance the generalizability of the results: Hong Kong Island (Pamela Youde Nethersole Eastern Hospital); Kowloon (Princess Margaret Hospital, Kwong Wah Hospital and Queen Elizabeth Hospital); and the New Territories (Prince of Wales Hospital). The study was initiated in March 2016 and continues until the end of 2017. Figure 1 is a Consort flow diagram of the progress through the phases of a parallel randomised trial of the intervention and control group.

### Randomisation and strata

This study adopts a two-arm (birth ball and control) randomised intervention-control trial design with block randomisation, as this is the most robust method of minimising selection bias and strengthening statistical power. We further enhance statistical power by using stratified randomisation according to potential confounders of parity (nulliparous or non-nulliparous) and type of labour onset (spontaneous or induced) – two strata give four possible combinations. Allocation is concealed by using sequentially numbered opaque sealed envelopes (in four separate sets) that contain group assignments determined by computer-generated random sequences. A research officer gives the sealed envelope to the health care worker who is intended to carry out the intervention. No intervention if subject is assigned to control.

### Sample size

Data to estimate sample size is only available from two randomised trials [13, 14]. Both trials have high risks of bias that likely lead to an overestimated effect size (Cohen’s d 0.90–1.34) in labour pain relief by birth ball, measured by visual analogue scale (VAS).

Without existing rigorous research enabling more accurate estimation of effect size, we re-calculated sample size with a conservative Cohen’s d of 0.25 in pain relief measured by VAS, alpha 0.05 and a power of 0.8. This was calculated using software G*Power (version 3.1.9.2). In order to have an equal number of participants in intervention and control groups, participants of each stratified block are further amended as 64 in each stratum. This gives a sample size of 512.

### Inclusion & exclusion criteria

For safety reason, pregnant women who are admitted to the hospital for labour and vaginal birth will not be considered unless they meet all the inclusion criteria. 1) Chinese women able to provide informed consent; 2) singleton pregnancy in cephalic presentation planned for vaginal birth; 3) gestational age of 37 to 42 weeks admitted into a single room in labour unit, either in spontaneous active labour and the second stage not imminent, or for induction of labour; 4) uncomplicated past obstetrical and antenatal history enabling them to be under independent midwifery care.

The exclusion criteria are 1) Multiple pregnancy; 2) foetal mal-presentation contraindicating vaginal delivery; 3) complicated pregnancy requiring close monitoring and restricted mobilization; 4) Caesarean delivery planned for this pregnancy; 5) any of the contraindication to use birth ball listed for each hospital (slightly varies due to different setting).

### Intervention and control

We recognise that the use of a birth ball is a complex intervention that changes the women’s usual care in several ways that some may consider confounding factors. Research suggests that splitting complex non-pharmacological pain relief methods into specific and placebo components may not be meaningful and may even produce false negative results [17]. Other non-pharmacological methods may include warm compresses, massage and transcutaneous electrical nerve stimulation.

Women in the intervention group are first offered and taught how to use the birth ball, followed by other non-pharmacological and pharmacological options, upon the women’s requests and advice from midwives. The existence and nature of placebo and the specific purpose of the trial is not disclosed at enrolment similar to other trials [18, 19].

Midwives or physiotherapists with certified birth ball training (42–76 in trial hospitals) or who attended birth ball teaching classes, provide instructions on birth ball exercises and supervise the participants (and her labour support person if present) using a birth ball. Instructions on four groups of exercises adapted from the Taiwan trial [13] and local study with high satisfaction [9] are given: 1) sit on birth ball; 2) kneel and lean forwards to hug the birth ball with both arms; 3) squat down; 4) variations of the above exercises together with a labour support person. Due to time constraints, group 1 and 2 are taught first and, if there is limited time, group 3 and 4 are left out. Women are suggested to use the birth ball for a minimum of 15 min in the beginning, then subsequently according to their own preferences of posture, type of exercise and pattern.

Women in the control group receive the usual midwifery care, without the midwives actively teaching and proactively suggesting women the use of the birth ball. Other non-pharmacological pain relief methods may include warm compresses, massage and transcutaneous electrical nerve stimulation. For ethical reasons, women in the control group can use the birth ball if they specifically request it. Their data remains in the control arm in intention-to-treat analysis.

### Outcome measurements

A Cochrane systematic review reported a list of core outcomes for labour pain control, agreed through a wide consultation with researchers and consumer groups [1]. These outcomes include many important clinical and patient-relevant measures relating to the mother and baby. The data is collected from participants’ medical record and questionnaire. A summary of the primary outcomes and corresponding measurements is listed in Table 1.

**Table 1:** Summary of primary outcomes and measurements

Primary outcomes	Measurements
1. Pain intensity	Visual analogue scale (VAS)
2. Satisfaction with pain relief	American Pain Society – Patient Outcome Questionnaire–Modified (APS-POQ Modified)
3. Sense of control in labour	29-item Chinese validated Labour Agentry Scale (c-LAS)
4. Satisfaction with childbirth experience	Six Simple Questions (SSQ)

#### Primary outcomes

A simple and rapid visual analogue scale (VAS) of a 10-cm horizontal line, subdivided evenly with marks. Scores range from 0 (no pain) to 10 (extreme pain) are rounded to the nearest 0.1 cm, based on the women’s perceptions of pain at the time of assessment and during the peak of the last contraction at various time points: 1) on admission; 2) pre- and post-analgesia; 3) during labour, minimally every 2–4 h according to the phase of labour; 4) within 2 h of childbirth. This scale is the most widely used pain assessment scale in other birth ball studies [8–10, 13, 14]. Other primary outcomes are:

- Satisfaction with pain relief

- Modified satisfaction subscale from the American Pain Society – Patient Outcome Questionnaire (APS-POQ Modified) [20] is a 6-point scale (from ‘very dissatisfied’ to ‘very satisfied’) assesses women’s overall satisfaction of labour. The items regarding physicians are excluded.
- Additional questions on the willingness to use different analgesic methods in their next pregnancy and the most satisfied non-pharmacological method.

- Sense of control in labour

A 29-item Chinese validated Labour Agentry Scale (c-LAS) [21, 22] is used. It has subjective statements such as ‘I felt confident’ and ‘I felt defeated’. Scores are given on a 7-point Likert scale (from ‘all the time’ to ‘almost never or never’).

- Satisfaction with childbirth experience

Six Simple Questions (SSQ) [23, 24] assesses women’s satisfaction with care during childbirth. Items are scored on a 6-point scale (from ‘strongly disagree’ to ‘strongly agree’), giving an overall score.

#### Secondary outcomes

Other data we collect as secondary outcomes include effect (negative) on mother-baby interaction [25, 26], breastfeeding practices, delivery that require assisted vaginal birth and/ or Caesarean section, adverse effects of mother and infant, admission to special care baby unit or neonatal intensive care unit, five-minute Apgar score, and infant outcomes at longer term follow-up.

### Demography and obstetrics data

Mothers’ demographic information is collected, such as maternal age, weight, height, socio-economic status, educational level, occupation, and marital status. Paternal age, occupation, and educational level are also noted. Other relevant obstetrical confounding factors of mothers on admission for labour – including parity, weeks of gestation, duration of labour prior to admission, degree of cervical dilation on admission and frequency of uterine contractions – are documented. During labour, information on any need for labour augmentation, foetal head position and status of amniotic fluid, are collected from medical records. Required infant data includes birth weight, gender, birth date, and body length. All the above information are recorded in the staff record form.

### Statistical methods

Baseline characteristics of the two groups are compared by an ANOVA test for continuous variables and χ [2]-test for categorical variables. The primary outcomes are differences in the mean change of VAS pre- and post-intervention between birth ball and other control methods first on the list, and differences in total scores of APS-POQ modified, c-LAS and SSQ between groups. Secondary measures are differences in c-PBQ score and mode of delivery (assisted vaginal and caesarean); differences in proportions of breastfeeding, adverse effects, admission to special care baby unit or neonatal intensive care unit, five-minute Apgar score less than seven; and differences in cost.

To control for potential cluster effect, (e.g., participants by certain midwives) we will use a linear mixed model where the midwife is treated as a random effect. The basic model is a random intercept model with compound symmetry as the covariance structure. The data fitness is explored using alternative models, e.g., using different covariance structures, will be compared by the restricted maximum likelihood.

Intention-to-treat analysis is adopted and performed by researchers masked to subjects’ group allocation. A two-sided *P* value of 0.05 or less is considered statistically significant. Objective measures and clinical outcomes are validated independently by two researchers. To minimise missing data, research officers ensure complete data collection prior to subjects’ hospital discharge and remind subjects that a follow-up assessment takes place at 6 to 8 weeks post-partum.

## Discussion

This study draws data from five different sites across Hong Kong, which will increase the generalizability of results and enable easier systematic adoption of the intervention in other non-trial units. With the large sample size, single-blinded and stratified randomisation design, the results we have will provide reliable data. We are using stratified randomisation according to potential confounders of parity (nulliparous or non-nulliparous) and type of labour onset (spontaneous or induced) – two strata give four possible combinations.

Taking ethical concerns into consideration, we are not forbidding women in the control group to use the birth ball if they request them, and vice-versa; we will not forcefully limit their other pain relief options. All the subjects from control group will remain in the control arm in the intention-to-treat analysis. This is regarded as a major limitation of the study that is not likely to be solved. Furthermore, since we aim to examine the effectiveness of the birth ball intervention as a whole in real clinical settings, these possible confounders are not intentionally separated out, as they are integral parts of birth ball application and implementation. Different hospitals have different cultures and settings, and hence this can lead to deviation in practice and therefore bias. To standardise the research protocol and administering of the intervention, meetings and training are given to the midwives at all study sites before and periodically during recruitment. There will be subgroup analysis in each hospital.

Other limitations of this study include the concomitant use of other non-pharmacological pain relief methods during labour. It is common for pregnant women to use several analgesic methods at the same time. Although the time and method of all the analgesic methods are noted, the outcome effects of using a birth ball would become difficult to measure. Besides, in a busy antenatal and labour ward, the completion of research data by midwives and staff would be less than expected. Our researchers follow-up each participant and ensure all the relevant information from the medical record is reviewed. Although it would be impossible to blind the midwives or physiotherapists who teach birth ball, all the researchers will not be disclosed the case or control status of participants in the ward and during data analysis.

Currently, birth balls are widely adopted in the delivery units in public and private hospitals in Hong Kong. Results from this study provide rigorous scientific evidence for policy development. If the results are favourable, it will facilitate systematic implementation to promote birth ball use for women in labour. Additionally, if proven effective and safe for mothers and babies, this simple non-invasive and non-pharmacological method can be used in delivery suites around the world, especially in low-resource settings at low cost to the healthcare system. Study results will be disseminated in peer-reviewed journals, academic and press conferences, and community conventions.

This study will be the largest RCT on birth ball use for pregnant women in the world, with a total of 512 subjects. Our primary research questions include the effect of using the birth ball on pain intensity, satisfaction with pain relief and childbirth experience, and sense of control in labour. Results will provide rigorous scientific evidence for clinical practice and policy development.

## Abbreviations

APS-POQ Modified: Modified satisfaction subscale from the American Pain Society – Patient Outcome Questionnaire–Modified
ChiCTR: Chinese Clinical Trial Register
c-LAS: Chinese validated Labour Agentry Scale
RCT: Rrandomised controlled trial
SSQ: Six Simple Questions
VAS: Visual analogue scale
